# Over 600 million people aged 18–65 will have headache tomorrow: global 1-day prevalence and recall bias from a meta-analysis of individual participant data (N = 38,512) from the general populations of 15 countries

**DOI:** 10.1186/s10194-026-02359-2

**Published:** 2026-05-25

**Authors:** Andreas Kattem Husøy, Shengyuan Yu, Ruozhuo Liu, Akbar A. Herekar, Bilal Ahmed, Arif D. Herekar, Callixte Kuate Tegueu, Anastase Dzudie Tamdja, Annick Mélanie Magnerou, Najib Kissani, Latifa Adarmouch, Thierry Adoukonou, Mendinatou Agbetou, Oyéné Kossi, Mehila Zebenigus, Redda Tekle-Haimanot, Dawit K. Worku, Girish N. Rao, Girish B. Kulkarni, Gopalkrishna Gururaj, Mohammed Al Jumah, Ali M. Al Khathaami, Suliman Kojan, Guiovanna Quispe, Carlos Palomino-Diaz, Youssoufa Maiga, Robert P. Cowan, Mattias Linde, Ajay Risal, Debashish Chowdhury, Krishnan Anand, Ashish Duggal, Otgonbayar Luvsannorov, Dorjkhand Baldorj, Selenge Enkhtuya, Ilya Ayzenberg, Zaza Katsarava, Gretchen L. Birbeck, Daiva Rastenytė, Lars Jacob Stovner, Timothy J. Steiner

**Affiliations:** 1 NorHead, Department of Neuromedicine and Movement Science, NTNU, Norwegian University of Science and Technology, Edvard Griegs gate, Trondheim, Norway; 2 Department of Neurology and Clinical Neurophysiology, St Olavs University Hospital, Trondheim, Norway; 3 Department of Neurology, Chinese PLA General Hospital, Beijing, China; 4 Headache Research Foundation of Pakistan, Karachi, Pakistan; 5 Department of Anesthesiology and Perioperative Medicine, Medical College of Georgia, Augusta, GA USA; 6 Department of Emergency Medicine, Manchester Royal Infirmary, Manchester University Foundation Trust, Manchester, UK; 7 Department of Neurosciences, Baqai Medical University, Karachi, Pakistan; 8 Department of Neurology, Douala Laquintinie Hospital, Douala, Cameroon; 9 Faculty of Medicine and Biomedical Sciences, University of Yaoundé, Yaoundé, Cameroon; 10 Department of Internal Medicine, Douala General Hospital, Douala, Cameroon; 11 Clinical Research Education, Networking and Consultancy (CRENC), Yaoundé, Cameroon; 12 Laboratory of Clinical and Experimental Neuroscience, Faculty of Medicine, Cadi Ayyad University, Marrakesh, Morocco; 13 Department of Neurology, Centre Hospitalier Universitaire Mohammed VI, Marrakesh, Morocco; 14 Community Medicine and Public Health Department, Bioscience and Health Research Laboratory, Faculty of Medicine, Cadi Ayyad University, Marrakech, Morocco; 15 Department of Neurology, University of Parakou, Parakou, Benin; 16 Department of Neurology, School of Medicine, College of Health Science, Addis Ababa University, Addis Ababa, Ethiopia; 17 Department of Internal Medicine, School of Medicine, College of Health Science, Addis Ababa University, Addis Ababa, Ethiopia; 18 Pulmonary Disease and Critical Care Medicine, Henry Ford Providence Southfield Hospital, Michigan State University College of Human Medicine, Southfield, MI USA; 19 Centre for Public Health, National Institute of Mental Health and Neuro Sciences (NIMHANS), Bangalore, India; 20 Department of Clinical Neurosciences, National Institute of Mental Health and Neuro Sciences (NIMHANS), Bangalore, India; 21 King Abdullah International Medical Research Centre, Riyadh, Saudi Arabia; 22 King Saud Bin Abdulaziz University for Health Sciences, Riyadh, Saudi Arabia; 23 InterHealth Hospital, Riyadh, Saudi Arabia; 24 King Abdulaziz Medical City, Riyadh, Saudi Arabia; 25 Neurology Service, Hospital Luis Negreiros Vega, Callao, Peru; 26 Department of Neurology, Gabriel Touré Teaching Hospital, Bamako, Mali; 27 Faculty of Medicine, University of Technical Sciences and Technologies, Bamako, Mali; 28 Department of Neurology and Neurosciences, Stanford University School of Medicine, Stanford, CA USA; 29 Regional Migraine Unit, Sahlgrenska University Hospital, Gothenburg, Sweden; 30 Department of Psychiatry, Kathmandu University School of Medical Science, Katmandu, Nepal; 31 GB Pant Institute of Postgraduate Medical Education and Research, New Delhi, India; 32 All India Institute of Medical Sciences, New Delhi, India; 33 Department of Neurology, Atal Bihari Vajpayee Institute of Medical Sciences and Dr RML Hospital, New Delhi, India; 34 Department of Neurology, Mongolian National University of Medical Sciences, Ulaanbaatar, Mongolia; 35 Department of Neurology, Buyant Onoshlogoo Clinic, Ulaanbaatar, Mongolia; 36 Reflex Neurology Hospital, Ulaanbaatar, Mongolia; 37 Department of Neurology, St. Josef-Hospital, Ruhr-University Bochum, Bochum, Germany; 38 Centre of Neurology, Geriatric Medicine and Early Rehabilitation, Evangelical Hospital, Unna, Germany; 39 Medical Faculty, University of Essen, Essen, Germany; 40 Neurology Research Office, University Teaching Hospitals, Lusaka, Zambia; 41 Department of Neurology, University of Rochester, Rochester, NY USA; 42 Lithuanian University of Health Sciences, Kaunas, Lithuania; 43 Department of Neurology, University of Copenhagen, Copenhagen, Denmark; 44 Division of Brain Sciences, Imperial College London, London, UK

**Keywords:** Migraine, Probable migraine, Definite migraine, Tension type headache, Probable tension type headache, Definite tension type headache, Medication-overuse headache, Global Burden of Disease (GBD), Meta-analysis, Individual participant data, Population-based study, 1-day prevalence, HARDSHIP questionnaire, Global Campaign against Headache

## Abstract

**Background:**

Our recent estimate of the global 1-year prevalence of headache among those aged 18–65 years was 65%: considerably higher than previous estimates, but based solely on high-quality epidemiological data derived from a large population-based sample. Here we present complementary estimates of 1-day prevalence.

**Methods:**

We performed a meta-analysis of individual participant data from cross-sectional surveys among population-representative samples (age range 18–65 years) from 15 countries and all world regions. All used the Headache-Attributed Restriction, Disability, Social Handicap and Impaired Participation (HARDSHIP) questionnaire, including the question “did you have a headache yesterday?”, from which 1-day prevalence was determined. An algorithmic process applying modified ICHD criteria yielded separate estimates for migraine, tension-type headache (TTH) and probable medication-overuse headache (pMOH: the association of headache on ≥ 15 days/month and medication overuse). We analysed associations with age, gender and country-income level, and adjusted prevalence estimates for these factors. We calculated predicted 1-day prevalence from 1-year prevalence and reported headache frequency.

**Results:**

Among the 38,512 participants, females (53.4%) and participants from low- (17.1%) or lower-middle-income countries (64.6%) were overrepresented, but age distribution fairly matched that of the world. Overall, 13.7% (95% CI: 13.3–14.0) reported headache yesterday, females (17.1% [16.6–17.6]) more than males (9.7% [9.3–10.2]). Migraine was the most common headache type yesterday (6.0% [5.8–6.3]), followed by TTH (4.1% [3.9–4.3]) and pMOH (2.3% [2.2–2.5]). One-day headache prevalence was higher in low/lower-middle-income countries (13.9% [13.6–14.3]) than in high/upper-middle-income countries (12.4% [11.6–13.2]). Predicted 1-day prevalence (10.9% [10.7–11.1]) was considerably lower than observed 1-day prevalence (13.7% [13.3–14.0]), although not among those with pMOH (3.1% [3.0-3.3] versus 2.3% [2.2–2.5]). Adjusted for age, gender and country-income level, global 1-day prevalence estimates were 13.1% (12.8–13.5) for any headache, 5.7% (5.5–5.9) for migraine, 3.9% (3.7–4.1) for TTH and 2.4% (2.3–2.6) for pMOH, with 1.0% undiagnosed.

**Conclusion:**

Assuming yesterday was no different from any other day, an estimated 13.1% (*N* = 641,900,000) of the world’s population aged 18–65 years will have headache tomorrow; almost half will be migraine. People with migraine or TTH underestimate the frequency of headache episodes. Since headache-attributed burden is usually estimated from recalled frequency over 1–12 months, this also may be underestimated.

**Supplementary Information:**

The online version contains supplementary material available at 10.1186/s10194-026-02359-2.

## Background

Our recent meta-analysis of 1-year headache prevalence among the world’s population aged 18–65 years (effectively those of working age) found headache disorders, including migraine, tension-type headache (TTH) and probable medication-overuse headache (pMOH), to be more prevalent than previous estimates had suggested [1]. From our findings, we expect two thirds (65%) of this population to experience headache during the coming year: 25% will have migraine, over 33% TTH and 4% pMOH [1]. Our meta-analysis was of individual participant data (IPD) from the HARDSHIP (Headache-Attributed Restriction, Disability, Social Handicap and Impaired Participation) adult database [2], from which we selected only those studies that had gathered samples representative of the general population (within the age range 18–65 years) and applied the same, standardized methodology [3, 4].

Built into this methodology was recognition of the unreliability of recall – usually over 1–12 months, upon which cross-sectional surveys depend. Recall error inevitably increases with greater recall period [5]. With this in mind, all surveys contributing to the database incorporated enquiry into headache yesterday (HY: headache on the day preceding interview), providing data expected to be free from recall error and from which estimates could be made of 1-day headache prevalence. This relatively novel measure can be very useful for estimating population burden when the sample is large enough (1-day prevalence is obviously much lower than 1-year prevalence) [6].

Our primary aims here, again by meta-analysis of IPD from the HARDSHIP database, were to estimate 1-day headache prevalence in the world’s population aged 18–65 years, and investigate its associations with age, gender and country-income level. But, notably, this estimate can be directly compared with predicted 1-day prevalence (calculated from 1-year prevalence and reported headache frequency), offering insight into the direction and magnitude of recall error. Obtaining this was our secondary but also important aim.

## Methods

The methods of this study were similar to those of our previous study of 1-year prevalence [1]. A short summary follows.

### Ethics

Ethics approvals and consents from participants in each contributing study had been obtained in accordance with the Declaration of Helsinki [7] and local requirements [8–23].

### Study design and data acquisition

This was a meta-analysis of IPD from 15 of the 24 countries (16 samples) currently in the adult HARDSHIP database [2], being representative of the general populations aged 18–65 years of Benin [8, 24], Cameroon [9, 25], China [10, 26], Ethiopia [11, 27], India (North Capital Region/Delhi [12, 28] and Karnataka State [13, 29]), Lithuania [14, 30], Mali [15] Mongolia [16, 31], Morocco [17, 32], Nepal [18, 33], Pakistan [19, 34], Peru [20, 35], Russia [21, 36], Saudi Arabia [22, 37] and Zambia [23, 38]. Supplementary Table 1 shows the details of all samples in the database.

Except for a necessary adaptation in one, each contributing study used the same, standardized methodology [3, 4]. All were cross-sectional surveys using randomized cluster sampling to be representative of the general population. During unannounced visits to random households within each cluster, one randomly selected adult aged 18–65 years was interviewed face-to-face. In Saudi Arabia, where culture precluded household visits, participants were contacted by cellphone, through random digit dialling [22].

The samples from Luxembourg, Netherlands and Spain that were included in our previous study of 1-year prevalence [1] were excluded from the present study. This was because engagement with participants was through self-administered questionnaires, a potential source of bias: these were less likely to be completed on days with headache but commensurately more likely to be completed on the first headache-free day after a headache episode, spuriously elevating estimated 1-day prevalence.

All interviews used the HARDSHIP questionnaire [4] translated into the local language(s) following a standardized protocol [39]. The questionnaire included demographic enquiry (age and gender) and a diagnostic module based on whichever version of the International Classification of Headache Disorders (ICHD) [40–42] was current at the time. The focus of each study was on migraine, TTH and pMOH.

### Data analysis

#### Headache diagnoses

All participants reporting headache during the preceding year were asked the diagnostic questions. Diagnoses were made algorithmically, through a process previously described in detail [1]. We applied neutral or conservative imputation rules in the case of missing data, these having the effect of preferring probable rather than definite diagnoses [1].

The algorithm first identified pMOH (the combination of headache on ≥ 15 days/month and reported acute medication overuse). For countries where triptans and/or compound analgesics were readily available (Lithuania, Morocco, Russia and Saudi Arabia), we set the threshold for medication overuse at 10 days/month, and elsewhere (Benin, Cameroon, Ethiopia, India, Mali, Mongolia, Nepal, Pakistan, Peru and Zambia) at 15 days/month. We had no information on medication consumption for the sample from China.

To all remaining participants (with the exception of those in Mali), the algorithm assigned a diagnosis according to their symptoms (of the most bothersome headache when more than one headache type was reported). In accordance with ICHD [40–42], the following hierarchy was applied: definite migraine, definite TTH, probable migraine, probable TTH. Headache fulfilling the criteria for none of these was recorded as *unclassified*. Definite and probable estimates were combined for migraine and TTH to give overall estimates of each headache type.

The study in Mali was of all headache and its attributed burden, without recording data for identifying headache type [15]: in this sample (*n* = 2,105), we could diagnose only pMOH.

#### One-day prevalence

Participants were asked whether they had experienced HY, with 1-day prevalence of any headache estimated from the responses to this question. This enquiry did not include diagnostic questions: with regard to the headache types included here, ICHD criteria apply to these as disorders, not to single episodes. Participants diagnosed with pMOH and reporting HY were assumed to have pMOH yesterday. Otherwise, diagnoses of HY could be inferred in those reporting only one headache type, and in those responding that HY was of the same type as their most bothersome headache (which had been diagnosed). In those remaining, including those whose most bothersome headache was unclassified, HY was recorded as *undiagnosed*.

#### Statistics

We used proportions (%), means and standard deviations (SDs) to describe sample demographics.

Prevalence estimates were reported as proportions (%) with 95% confidence intervals (CIs). Information from The World Bank was used to classify countries by income level at the times of data collection [43]. High- and upper-middle-income countries were grouped together, as were low- and lower-middle-income countries, since we had limited samples from each income level.

Predicted 1-day prevalence was calculated by multiplying 1-year prevalence and mean reported headache frequency in the preceding month (days/month; possible range 0–30) and dividing by 30. The uncertainties of both estimates (1-year prevalence and mean headache frequency) were propagated to generate 95% CIs (mean±[1.96*SE]) of the predictions, with standard error (SE) calculated from the formula:

$$\begin{gathered} \:\frac{{S{E_{prediction}}}}{{prediction}} = \hfill \\ \sqrt {{{\left( {\frac{{S{E_{prevalence}}}}{{prevalence}}} \right)}^2} + {{\left( {\frac{{S{E_{frequency}}}}{{mea{n_{frequency}}}}} \right)}^2}} \hfill \\ \end{gathered} $$

Age-, gender- and country-income level-adjusted prevalence estimates were calculated using the most recent (2024) age and gender distributions within each of the four income levels [44]. We used RStudio version 2023.06.2 + 561 [45] for all analyses.

**Table 1:** Characteristics of the sample compared to those of the world population aged 18–65 years

	Sample			World [44] (billions)
	Overall	Males^a^	Femalesa	
**Sample size**, n (%)	38,512 (100)	17,946 (46.6)	20,563 (53.4)	4.90; 50.6% males
**Age**^**b**^, mean±SD	37.4±13.0	37.8±13.1	37.1±12.9	-
18–25, n (%)	8,722 (22.6)	3,982 (22.2)	4,738 (23.0)	0.98 (20.0)
26–35, n (%)	10,235 (26.6)	4,533 (25.3)	5,702 (27.7)	1.19 (24.3)
36–45, n (%)	8,996 (23.4)	4,334 (24.2)	4,661 (22.7)	1.08 (22.0)
46–55, n (%)	6,047 (15.7)	2,894 (16.1)	3,153 (15.3)	0.92 (18.9)
56–65, n (%)	4,495 (11.7)	2,197 (12.2)	2,298 (11.2)	0.73 (14.8)
**Income level**				
High, n (%) Saudi Arabia, n	2,316 (6.0) 2,316	1,442 (8.0) 1,442	874 (4.3) 874	0.89 (18.2)
Upper-middle, n (%) Lithuania, n Russia, n Peru, n	4,746 (12.3) 572 2,025 2,149	2,261 (12.6) 236 960 1,065	2,485 (12.1) 336 1,065 1,084	1.84 (37.6)
Lower-middle, n (%) China, n Benin, n Cameroon, n India, Karnataka, n India, Delhi NCR, n Mongolia, n Morocco, n Pakistan, n Zambia, n	24,860 (64.6) 5,041 2,400 3,100 2,329 2,066 2,041 2,575 4,223 1,085	11,334 (63.2) 2,561 1,231 1,407 1,141 737 812 1,038 1,957 450	13,523 (65.8) 2,480 1,169 1,693 1,188 1,329 1,229 1,535 2,265 635	1.85 (37.8)
Low, n (%) Ethiopia, n Mali, n Nepal, n	6,590 (17.1) 2,385 2,105 2,100	2,909 (16.2) 1,057 991 861	3,681 (17.9) 1,328 1,114 1,239	0.31 (6.3)

^a^Three participants were missing information on gender; ^b^17 participants were missing information on age

## Results

### Description of the sample

Among the sample of 38,512 participants, females (53.4% versus 49.4%) were slightly overrepresented and those from low (17.1% versus 6.3%) or lower-middle-income countries (64.6% versus 37.8%) were more substantially overrepresented in comparison with the world’s population aged 18–65 years (Table 1) [44]. Mean age in the sample was 37.4 years (SD = 13.0 years), similar among males and females and fairly matching the world distribution (Table 1) [44].

### 1-day prevalence

HY was reported by 5,263 participants, an observed 1-day prevalence of any headache of 13.7% (95% CI: 13.3–14.0) (Table 2). According to inferred diagnoses, most prevalent was migraine (6.0% [5.8–6.3]), followed by TTH (4.1% [3.9–4.3]) and pMOH (2.3% [2.2–2.5]), with 1.0% (7.1% of all HY) undiagnosed.

HY was more common among females (17.1% [16.6–17.6]) than males (9.7% [9.3–10.2]): this was true for all headache types, while, notably, migraine yesterday was almost twice as common among females and pMOH yesterday almost three times as common.

**Fig. 1:**
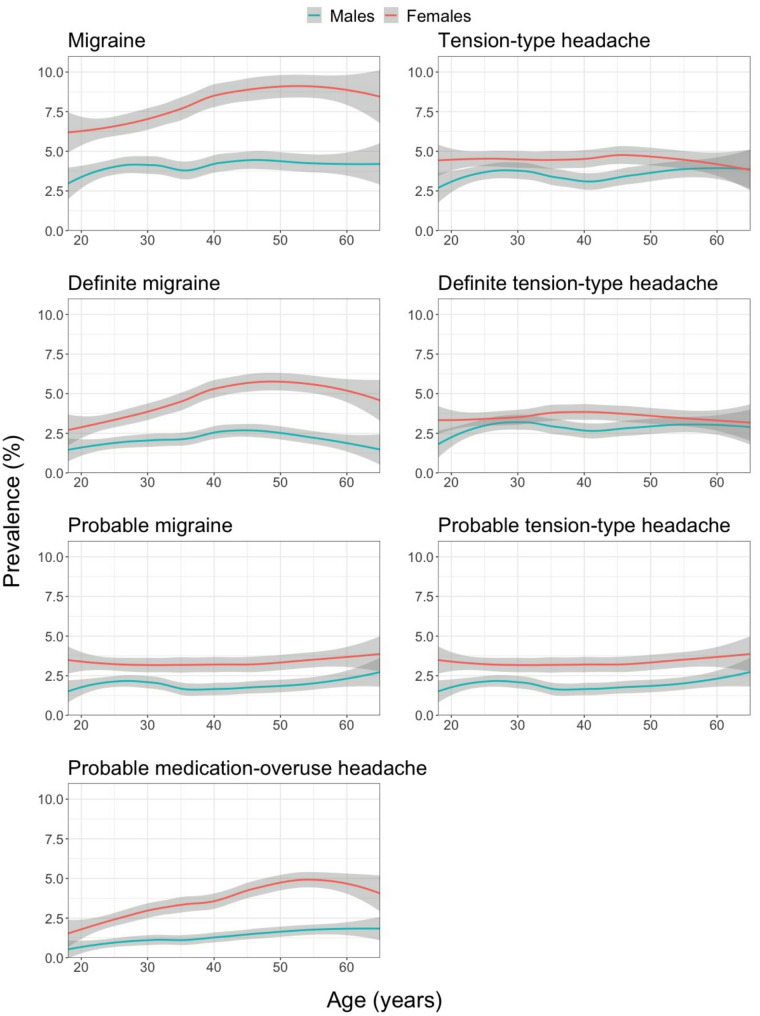
Observed 1-day prevalence of each headache type in the total sample by age and gender (grey areas represent 95% confidence intervals)

Table 2 shows 1-day prevalence related to age for the different headache types. Figure 1 illustrates this better, with gender-specific plots. For migraine there was a slight inverted U-shape for females, with the highest prevalence in the 50s (about 9%), driven primarily by definite migraine. Among males, migraine prevalence varied much less with age, but also here there was a slight inverted U-shape for definite migraine. Probable migraine changed minimally with age. TTH prevalence (definite and probable) remained relatively stable with age. Among females, pMOH peaked in the 50s at around 5%, followed by a decline; this was not so among males, who showed a steady increase plateauing at 2% in the 50s and 60s.

HY was slightly more common in low/lower-middle-income countries (13.9% [13.6–14.3]) than in high/upper-middle-income countries (12.4% [11.6–13.2]) (Table 2).

**Table 2:** Observed 1-day prevalence (% [95% confidence interval]) of headache in the total sample

	Any headache	Migraine^d^			Tension type headache^d^			pMOH^e^	Undiagnosed^d^
		Total^c^	Definite	Probable	Total^c^	Definite	Probable		
**Overall**	13.7 [13.3–14.0]	6.0 [5.8–6.3]	3.4 [3.2–3.5]	2.7 [2.5–2.8]	4.1 [3.9–4.3]	3.2 [3.1–3.4]	0.8 [0.7–0.9]	2.3 [2.2–2.5]	1.0 [0.9–1.1]
Male	9.7 [9.3–10.2]	4.0 [3.7–4.3]	2.1 [1.9–2.3]	1.9 [1.7–2.1]	3.6 [3.3–3.8]	2.9 [2.6–3.1]	0.7 [0.5–0.8]	1.2 [1.1–1.4]	0.7 [0.6–0.8]
Female	17.1 [16.6–17.6]	7.7 [7.4–8.1]	4.4 [4.2–4.7]	3.3 [3.1–3.6]	4.5 [4.2–4.8]	3.5 [3.3–3.8]	1.0 [0.8–1.1]	3.3 [3.1–3.6]	1.3 [1.1-1.]
**Age (years)**									
18–25	11.6 [11.0-12.3]	5.1 [4.6–5.5]	2.5 [2.1–2.8]	2.6 [2.3-3.0]	4.0 [3.5–4.4]	3.0 [2.6–3.4]	1.0 [0.8–1.2]	1.3 [1.1–1.6]	1.1 [0.9-1.]
26–35	13.5 [12.8–14.1]	5.8 [5.4–6.3]	3.1 [2.8–3.5]	2.7 [2.4-3.0]	4.1 [3.7–4.5]	3.3 [3.0-3.7]	0.8 [0.6-1.0]	2.1 [1.8–2.4]	1.2 [1.0-1.5]
36–45	13.7 [13.0-14.4]	6.5 [6.0–7.0]	4.1 [3.6–4.5]	2.4 [2.1–2.8]	3.8 [3.4–4.2]	3.3 [2.9–3.7]	0.5 [0.4–0.7]	2.5 [2.2–2.8]	0.8 [0.6-1.]
46–55	15.8 [14.9–16.7]	6.6 [6.0-7.3]	3.9 [3.4–4.4]	2.7 [2.3–3.1]	4.4 [3.9-5.0]	3.3 [2.8–3.8]	1.1 [0.9–1.4]	3.4 [3.0-3.9]	0.9 [0.7–1.2]
56–65	15.1 [14.0-16.1]	6.6 [5.8–7.3]	3.5 [3.0-4.1]	3.1 [2.5–3.6]	3.9 [3.3–4.5]	3.2 [2.6–3.7]	0.7 [0.5-1.0]	3.2 [2.7–3.7]	1.0 [0.7–1.3]
**Income**									
high/upper-middle^a^	12.4 [11.6–13.2]	5.4 [4.8–5.9]	3.1 [2.8–3.4]	2.4 [2.0-2.7]	3.7 [3.2–4.1]	2.8 [2.4–3.2]	0.9 [0.6–1.1]	2.5 [2.1–2.8]	0.9 [0.7.-1.1]
low/lower-middle^b^	13.9 [13.6–14.3]	6.2 [5.9–6.5]	3.4 [3.2–3.7]	2.7 [2.5–2.9]	4.2 [3.9–4.4]	3.3 [3.1–3.5]	0.8 [0.7–0.9]	2.3 [2.1–2.5]	1.0 [0.9–1.2]

pMOH: probable medication-overuse headache; ^a^Lithuania, Russia, Peru and Saudi Arabia [43]; ^b^Benin, Cameroon, China, Ethiopia, India (Karnataka and Delhi regions), Mali, Mongolia, Morocco, Nepal, Pakistan and Zambia [41]; ^c^definite and probable combined; ^d^Mali sample excluded (the denominator for migraine, tension-type headache and undiagnosed headache [*N* = 36,407] is therefore different from the denominator for any headache [*N* = 38,512], and summed prevalences of each type do not exactly match the prevalence of any headache); ^e^China sample excluded (the denominator for pMOH [*N* = 33,471] is therefore different from the denominator for any headache [*N* = 38,512], and summed prevalences of each type do not exactly match the prevalence of any headache)

**Table 3:** Predicted versus observed 1-day prevalence of headache in the total sample

Headache type	1-year prevalence %±SE	Headache frequency (days/month) mean±SE	Predicted 1-day prevalence^a^ % [95% CI]	Observed 1-day prevalence % [95% CI]
Any headache	64.8±0.002	5.1±0.05	10.9 [10.7–11.1]	13.7 [13.3–14.0]
Migraine^b^	25.7±0.003	4.9±0.06	4.2 [4.1–4.4]	6.0 [5.8–6.3]
definite	13.1±0.002	5.3±0.09	2.3 [2.2–2.4]	3.4 [3.2–3.5]
probable	12.7±0.002	4.6±0.09	1.9 [1.9-2.0]	2.7 [2.5–2.8]
Tension-type headache^b^	33.2±0.002	3.4±0.04	3.8 [3.7–3.9]	4.1 [3.9–4.3]
definite	26.6±0.002	3.4±0.05	3.0 [2.9–3.1]	3.2 [3.1–3.4]
probable	6.6±0.001	3.6±0.10	0.8 [0.7–0.8]	0.8 [0.7–0.9]
Probable medication-overuse headache^c^	4.0±0.001	23.6±0.19	3.1 [3.0-3.3]	2.3 [2.2–2.5]

SE: standard error; CI: confidence interval; ^**a**^predicted from 1-year prevalence and mean headache frequency; ^b^Mali sample excluded (the denominator for migraine and tension-type headache [*N* = 36,407] is therefore different from the denominator for any headache [*N* = 38,512], and summed prevalences of each type do not exactly match the prevalence of any headache); ^c^China sample excluded (the denominator for pMOH [*N* = 33,471] is therefore different from the denominator for any headache [*N* = 38,512], and summed prevalences of each type do not exactly match the prevalence of any headache)

### Predicted versus observed 1-day prevalence

Table 3 shows the predicted and observed 1-day prevalences, the former significantly lower for any headache than the latter (10.9% [10.7–11.1] versus 13.7% [13.3–14.0]). Participants with migraine showed the largest discrepancy: 4.2% (4.1–4.4) versus 6.0% (5.8–6.3), with corresponding differences in both definite and probable migraine. A smaller discrepancy in TTH (3.8% [3.7–3.9] versus 4.1% [3.9–4.3]) was entirely accounted for by definite TTH. In contrast, predicted 1-day prevalence of pMOH (3.1% [3.0-3.3]) was higher than observed (2.3% [2.2–2.5]).

Notably, 47.0% of those with pMOH reported headache on 30 days/month. In seven of the contributing studies, response options included “every day”, which was selected by 53.3% of participants with pMOH in these studies.

### Age-, gender- and income-adjusted global estimates

Table 4 shows global age-, gender- and country-income-adjusted 1-day prevalences. An estimated 13.1% (12.8–13.5) of the world’s population aged 18–65 years had HY. Again according to inferred diagnoses, migraine was the most prevalent headache type (adjusted 1-day prevalence 5.7% [5.5–5.9], definite 3.1% [2.9–3.3], probable 2.6% [2.4–2.7]), followed by TTH (3.9% [3.7–4.1], definite 3.1% [2.9–3.2], probable 0.8% [0.7–0.9]) then pMOH (2.4% [2.3–2.6]).

**Table 4:** Age-, gender- and country-income level-adjusted global 1-day headache prevalence (% [95% confidence interval])

Headache type	Adjusted 1-day prevalence % [95% CI]
Any headache	13.1 [12.8–13.5]
Migraine^a^	5.7 [5.5–5.9]
definite	3.1 [2.9–3.3]
probable	2.6 [2.4–2.7]
Tension-type headache^a^	3.9 [3.7–4.1]
definite	3.1 [2.9–3.2]
probable	0.8 [0.7–0.9]
Probable medication-overuse headache^b^	2.4 [2.3–2.6]
Undiagnosed^a^	1.0 [0.9–1.1]

^a^Mali sample excluded (the denominator for migraine, tension-type headache and undiagnosed headache [*N* = 36,407] is therefore different from the denominator for any headache [*N* = 38,512], and summed prevalences of each type do not exactly match the prevalence of any headache); ^b^China sample excluded (the denominator for pMOH [*N* = 33,471] is therefore different from the denominator for any headache [*N* = 38,512], and summed prevalences of each type do not exactly match the prevalence of any headache)

### Allocation of diagnoses to HY

**Fig. 2:**
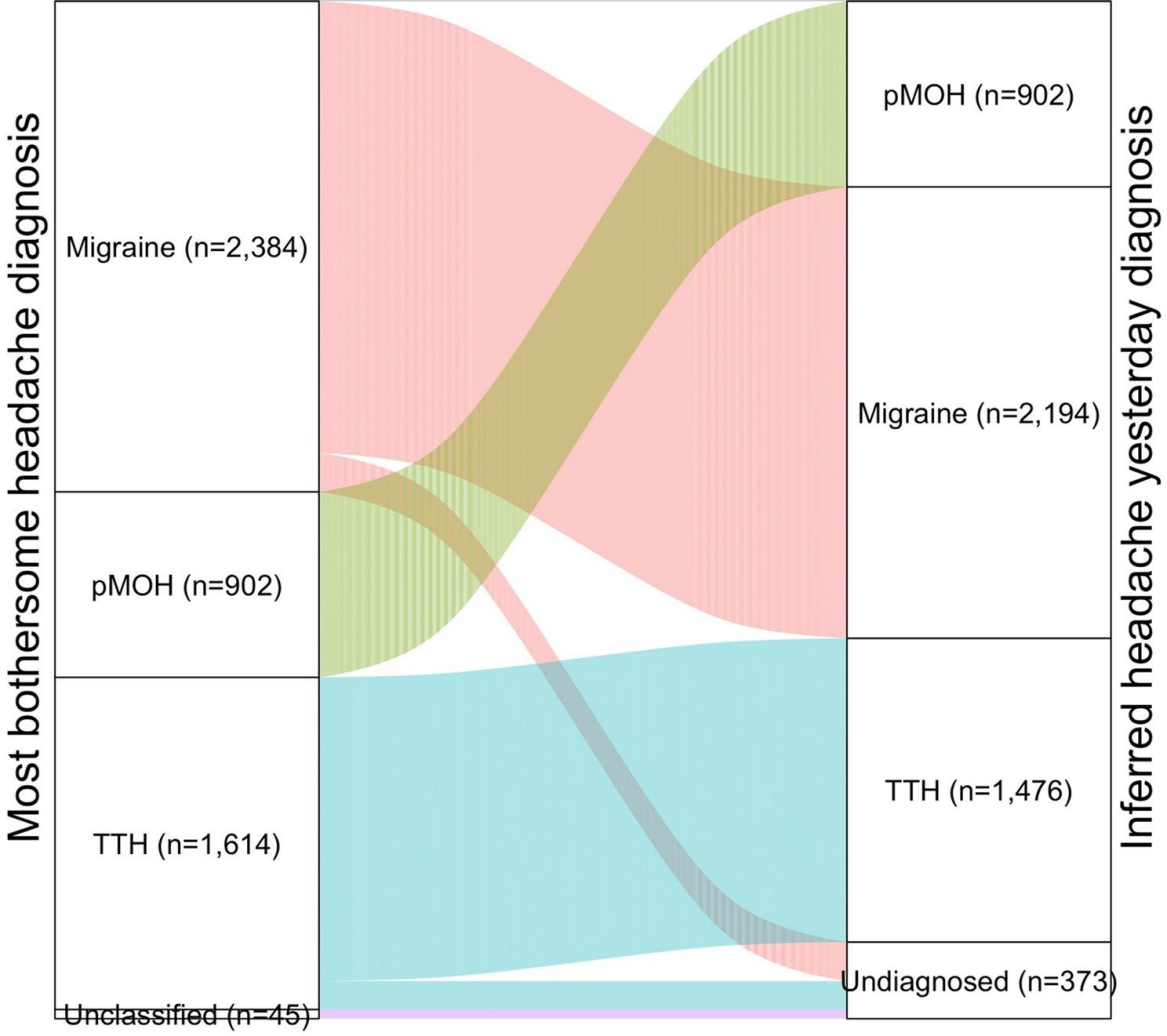
Sankey diagram showing how headache yesterday was classified diagnostically in relation to the diagnosed most bothersome headache (Mali sample excluded; in all other samples, headache yesterday was undiagnosed in participants reporting more than one headache type *and* that headache yesterday was not of the most bothersome type, and in those whose most bothersome headache was unclassified)

As noted, ICHD criteria could not be applied directly to HY, but diagnosis by inference was possible for most cases. Among participants diagnosed with pMOH, HY, when reported (*n* = 902), was assumed to be pMOH (see Methods). Of the other 4,043 participants with HY (Mali excluded, because of lack of diagnostic data), 3,654 (90.4%) reported only one headache type or that HY was the same as their most bothersome headache (with no gender-related differences). Accordingly, in these, a diagnosis for HY could be confidently inferred. Of the 2,384 with HY whose most bothersome headache was migraine, 190 (8.0%) reported more than one headache type *and* that HY was not the same as their most bothersome. In these, HY could not be diagnosed (Fig. 2). The corresponding proportion for TTH (*n* = 1,614) was 138 (8.6%).

## Discussion

In this meta-analysis of IPD (*N* = 38,512) from cross-sectional surveys of population-representative samples (aged 18–65 years) in 15 countries throughout the world, we found that 13.7% of participants reported headache on the day preceding the survey interview. Adjusting for age, gender and country-income level, we estimated a global 1-day prevalence among this age group of 13.1%. Assuming yesterday was no different from any other day, this indicates that approximately 642 million people (13.1% of the global population of 4.90 billion aged 18–65 years [44], who are effectively the working population) will have headache tomorrow. Of these, an estimated 5.7% (*n* = 279 million) will have migraine, 3.9% (*n* = 191 million) TTH and 2.4% (*n* = 118 million) pMOH, with 1.0% (*n* = 49 million) undiagnosed.

This meta-analysis applied the same methodology as our previous meta-analysis of IPD (*N* = 41,614) determining 1-year prevalence [1]. Some of the limitations of that study applied here. While we considered these limitations in detail in the earlier paper [1], we must briefly do so again here. An inherent and insurmountable limitation of all questionnaire-based headache surveys is the dependence on subjective reporting of headache characteristics in order to make headache diagnoses. This means language and culture might have influenced our estimates, although all interviews were conducted in the local languages, and all translations of the HARDSHIP questionnaire followed a standardized protocol [39]. Although all 16 samples (from 15 countries) included in this meta-analysis were generated through cluster-based random sampling, the countries themselves were selected opportunistically (and to some extent purposively and with regard to feasibility in order to fill the large data gap in low- and lower-middle-income countries) rather than randomly. This resulted in overrepresentation of lower-middle-income countries, necessitating adjustment of global estimates not only for age and gender but also for country income level.

One of the main limitations of this study, however, was not encountered in our previous study of 1-year prevalence: the lack of a full set of diagnostic questions regarding HY. The HARDSHIP questionnaire was constructed in alignment with ICHD, which, with regard to the headache types included here, apply criteria to these as disorders, not to single attacks [40–42]. To circumvent this limitation, we relied on the diagnoses of participants’ most bothersome headache (for which a full set of diagnostic questions had been asked). We inferred, with reasonable confidence, that the diagnosis of HY was the same whenever HY was reportedly of the same type as the most bothersome headache (92.1% of cases, with no gender-related difference). But HY could not be diagnosed among the 1.0% of participants (7.1% of the 13.7% reporting HY) whose most bothersome headache was itself unclassified, or was migraine or TTH but who reported HY to be of another type. According to statistical probability, most undiagnosed HY would have been migraine or TTH, with 1-day prevalences of migraine and TTH commensurately underestimated. We might, in a theoretical exercise, have assumed with quite high probability that, if HY was not of the same type as migraine (190 participants), then it was TTH, and vice versa (138 participants), increasing *observed* 1-day prevalence of migraine from 6.0% to 6.4% and of TTH from 4.1% to 4.6%. We did not do this, for two reasons. First, a single participant might have had more than one (sub)type of migraine or TTH, not only distinguishing between these but also regarding one as more bothersome than the other. Second, we recognised that secondary headaches such as headache associated with acute viral illness might have featured – and probably did – among HY that reportedly was not the same as the most bothersome type. The result is *conservative* estimates of 1-day prevalence of migraine and TTH.

Nearly half of HY was diagnosed inferentially as migraine (age- and gender-adjusted prevalence of 5.7%). This was not unexpected, given its relatively high 1-year prevalence (~ 25%, Table 3) and headache frequency (mean = 4.9 days/month; Table 3). TTH was more prevalent over 1 year (~ 33%; Table 3), but, with fewer headache days/month (mean = 3.4; Table 3), its 1-day prevalence was lower (3.9%). pMOH showed the opposite: a low 1-year prevalence (~ 4%; Table 3) but high frequency (mean = 23.5 days/month; Table 3), yielding a 1-day prevalence of 2.3%.

Our results underscore that migraine is the most burdensome of all headache types at population level, in alignment with most previous literature including the most recent findings of the Global Burden of Disease study (GBD) [46]. However, our finding that migraine yesterday was more prevalent than TTH yesterday is in conflict with one of our previous literature meta-analyses showing TTH (8.7%) to have a higher 1-day prevalence than migraine (7.0%) [6]. A limited number of studies (only five on migraine and four on TTH) contributed to that meta-analysis [6]. Furthermore, it included HARDSHIP studies that we excluded from the present analyses because of likely selection bias (e.g., clinic-based samples, or low participating proportions; see Supplementary Table 1). These factors probably explain the higher 1-day prevalence of any headache in that study (15.8%, with a wide 95% CI of 10.0–22.0 [6]) compared with ours (13.1% [12.7–13.4]).

The relationship between 1-day prevalence and age resembled what we found in our previous paper on 1-year prevalence [1]: small differences possibly explained by a variable association between age and headache frequency. Definite migraine yesterday (inferentially diagnosed) peaked in the 40s to 50s in both males and females; TTH and probable migraine (also inferred) varied little with age; pMOH peaked in the 50s in females and reached a plateau at the same age in males. HY of all headache types was more common among females. This, for TTH, was not the case with regard to 1-year prevalence [1], the discrepancy being due to higher headache frequency of TTH episodes among females than among males.

Country-income level had a modest but significant influence: HY was more common in low/lower-middle-income countries (13.9%) than in high/upper-middle-income countries (12.4%). This is in contrast with 1-year headache prevalence in both GBD, which shows a downward gradient from high- to low-income countries [47], and our previous paper (1-year prevalence of 71.4% in high/upper-middle-income countries, 63.0% in low/lower-middle-income countries [1]). Here we had limited representation from each income level (Table 1), in particular from high-income countries (only Saudi Arabia), and collapsing these into two levels lost granularity. These analyses should be interpreted with caution, but the evidence does not suggest a strong influence of country-income level on 1-day headache prevalence. If there were, it would, speculatively, be due to poor access to health care and preventative medications, but access to these medications appears limited in high-income countries also [48, 49].

An important finding for estimates of headache-attributed burden, and for health policy, was that people with headache underestimated the frequency of their headache episodes, observed 1-day prevalence being 13.7%, predicted (from 1-year prevalence and mean recalled headache frequency) being 10.9%. The magnitude of recall error is likely to increase with the length of the recall period [5], Currently, GBD relies on data from the past 1–12 months to calculate *time spent with headache* (from headache frequency and headache duration), a key metric used as a factor in their health-loss estimates for headache disorders. At population level, more time may be spent with headache than previously estimated, and future studies should include, and directly compare, population-level estimates of time spent with headache based on 1-year and 1-day data.

Notably, those with pMOH had a higher predicted (3.1%) than observed (2.3%) 1-day prevalence, in contrast to those with migraine or TTH, suggesting that a tendency to underestimate is associated with relatively low headache frequency whereas relatively high frequency leads to overestimation. This may not be true, however, across the frequency spectrum: the ratio between observed and predicted prevalence was greater for migraine (6.0/4.2 = 1.43) than for TTH (4.1/3.8 = 1.08), despite that people with migraine reported a higher mean headache frequency than those with TTH (4.9 versus 3.4 days/month). An element of self-fulfilment was possible: participants who were overusing medication and who overestimated headache frequency increased their likelihood of diagnosis with pMOH, but these were probably few (those whose “true” headache frequency was in the range of 12–14 days/month). Response options in seven of the contributing studies included “every day” (treated as 30 days/month in the analyses), which was selected by 53.3% of those with pMOH in these studies. This might have been an inviting option when frequency was very high, but in the other studies, where this option was not offered, 38.7% replied that headache occurred on 30 days/month. There was evidence of terminal digit preference (for 5 or 0), a form of rounding error more likely to be upwards than downwards. Otherwise, if there was a general tendency to overestimate headache frequency when it was high (> 15), this would not have led to diagnostic misclassification.

### Strengths and limitations

The strength of this meta-analysis that distinguishes it from other studies estimating the global prevalence of headache (as a prerequisite for population-level burden estimates) was the extremely short recall period of 1 day, essentially eliminating recall error. Other important strengths were the large number of participants (*N* = 38,512, of whom 5,263 reported HY) sampled from the general population, and the fact that all contributing studies used the same standardized methodology [3, 4]. Meta-analyses based on IPD rather than on aggregate data are considered the gold standard of meta-analytical approaches [50].

Some limitations have already been discussed. Even though HY prevalence was assessed without recall error, diagnoses of HY were inferred from data that did depend on recall. Diagnoses could not be inferred for 7.1% cases of HY, and these might have included secondary headaches. The 1-year prevalence of chronic secondary headaches other than MOH is low (< 1% [51]), but, as noted, causes such as acute viral infections might have been behind some cases of HY, most probably HY reportedly different from the most bothersome headache (such causes of occasional headache would rarely be considered most bothersome) but in some cases possibly identified as migraine or TTH. Nevertheless, for reasons explained, we believe our estimates of 1-day prevalence of migraine and TTH were conservative. The diagnosis of pMOH was based on the association of headache on ≥ 15 days/month with acute medication overuse, without evidence of causation (hence the term “probable MOH”). The threshold for medication overuse (10 or 15 days/month) was also set on a country level according to assumed access to acute medications, which might have led to mis-identification of pMOH in participants from countries with (theoretically) easy access to triptans or opioids that used only simple analgesics on 10–14 days/month. Only one diagnosis (of most bothersome headache) was allowed per participant, but this had little relevance to HY (multiple types of headache in one day were unlikely).

As already stated, prevalence studies are a prerequisite for population-level burden estimates but, without the latter, they provide limited information for health policy. These estimates will follow in further IPD meta-analyses using the HARDSHIP database [2].

## Conclusions

Assuming yesterday was no different from any other day, an estimated 13.1% of the world’s population aged 18–65 years (*N* = 641,900,000) will have headache tomorrow, among whom almost half (5.7%) will have an episode of migraine. An incidental but important finding was that people with migraine or TTH underestimate the frequency of their headache episodes. This has implications for studies of headache-attributed burden, which are usually dependent on symptoms recalled over the preceding 1–12 months.

## Electronic Supplementary Material

Below is the link to the electronic supplementary material.

Supplementary Material 1

## Data Availability

The original data are held at Norwegian University of Science and Technology, Trondheim, Norway. Anonymised data are available on request for academic purposes, in line with the policy of the Global Campaign against Headache.

## Abbreviations

CI: Confidence interval
HARDSHIP: Headache-Attributed Restriction, Disability, Social Handicap and Impaired Participation (questionnaire)
H15+: Headache on ≥ 15 days/month
HY: Headache yesterday
LTB: Lifting The Burden
MOH: Medication-overuse headache
NorHEAD: Norwegian Centre for Headache Research
pMOH: Probable MOH
SD: Standard deviation
SE: Standard error
TTH: Tension-type headache
